# An Uncommon Confluence: Splenic Lymphangioma and Appendiceal Mucinous Neoplasm

**DOI:** 10.7759/cureus.93804

**Published:** 2025-10-04

**Authors:** İlkay Çinar, Ilker Sengul, Ali Muhtaroğlu, Demet Sengul

**Affiliations:** 1 Pathology, Giresun University Faculty of Medicine, Giresun, TUR; 2 Endocrine Surgery and General Surgery, Giresun University Faculty of Medicine, Giresun, TUR; 3 General Surgery, Giresun University Faculty of Medicine, Giresun, TUR

**Keywords:** appendiceal mucinous neoplasm, appendix, lymphangioma, mucinous neoplasm, pathology, spleen, splenic lymphangioma, surgery

## Abstract

Splenic lymphangiomas, per se, are exceedingly rare, benign congenital malformations of lymphatic vessels, typically diagnosed in childhood or incidentally in adulthood. Appendiceal mucinous neoplasms (AMNs) are also infrequent, often asymptomatic, and usually discovered incidentally. To date, the co-occurrence of these two distinct primary lesions in the same patient has not been previously reported. We present the case of an 83-year-old Turkish female who underwent emergent abdominal surgery for an acute abdomen secondary to a strangulated left diaphragmatic herniation. Multiple perforations were identified in the transverse colon, accompanied by widespread ischemia involving the terminal ileum, cecum, and right colon. Upon laparotomy, a diaphragmatic defect measuring approximately 10 cm in diameter was identified in the left hemidiaphragm. The herniated abdominal viscera were meticulously reduced and examined, and multiple perforations were identified in the transverse colon, accompanied by widespread ischemia involving the terminal ileum, cecum, and right colon. An emergent splenectomy was performed due to severe ischemia and irreparable structural damage observed within the compromised hernia sac, with the resection of the terminal ileum, the ascending, and transverse colon. During the operative intervention, both a splenic lymphangioma and a low-grade AMN were incidentally discovered. The histological diagnosis was confirmed with factor VIII-related antigen (factor VIII-R antigen) immunohistochemical positivity. The patient, unfortunately, succumbed on the fifth postoperative day due to progressive multi-organ failure. This report documents the first known instance of the simultaneous identification of splenic lymphangioma and AMN as primary lesions in a single patient. This unique case underscores the critical importance for surgeons to remain vigilant for unexpected pathologies and highlights the necessity of a multidisciplinary approach in evaluating rare incidental findings for appropriate classification and follow-up.

## Introduction

Lymphangiomas, or similar benign congenital malformations of the lymphatic vessels, are of a rare occurrence. Approximately 90% of these cases are observed in the regions of the neck and axilla [1]. More infrequently, their presence is discerned within the mediastinum, the pulmonary organs, the liver, and the retroperitoneum. Indeed, isolated splenic involvement is exceedingly uncommon. Splenic lymphangioma is characterized by single or multiple thin-walled cystic spaces within the spleen, which are lined by lymphatic endothelium [2]. In most cases, ranging from 80 to 90%, diagnosis occurs during childhood; manifestation in adulthood is extremely rare. Smaller lesions often present asymptomatically, being detected by mere fortuity; whereas larger lesions may occasion nonspecific symptoms such as pain in the left upper quadrant, nausea, vomiting, loss of appetite, a palpable mass, or splenomegaly. Complications, though seldom, may yet ensue, including rupture, hemorrhage, hypersplenism, or portal hypertension [3]. The preoperative diagnosis of cystic masses within the spleen has proven difficult. Ultrasonography, computed tomography (CT), and magnetic resonance imaging (MRI) may serve to elucidate the cystic structure; however, a definitive diagnosis is established solely through histopathological examination. For therapeutic purposes, splenectomy is the preferred course of action, serving both for diagnostic confirmation and for the prevention of complications [4]. Appendiceal mucinous neoplasms (AMNs) comprise but 1 to 2% of appendectomy specimens, and fewer than 0.3% of all appendectomies. They are typically diagnosed in individuals aged between 50 and 60 years, showing no marked predilection for either gender. A considerable portion of these cases remain asymptomatic, yet some may present with symptoms akin to acute appendicitis. Furthermore, the preoperative diagnosis is fraught with difficulty, and such lesions are frequently discovered incidentally upon histopathological review. Should rupture occur, a risk of developing pseudomyxoma peritonei (PMP) does arise, necessitating scrupulous evaluation during surgical intervention [5,6]. Moreover, the patient exhibited a reduction in vital capacity, attributable to the pressure exerted on the lung by herniated organs in the left hemithorax. The surgical procedure was initiated using an open conventional approach, as it was imperative to ensure that intra-abdominal pressure remained at a low level. Of note, the extremely rare co-occurrence of splenic lymphangioma and AMN in the same patient has, to our knowledge, not been previously documented in the medical literature, making the presented case particularly noteworthy.

## Case presentation

Brief presentation

The patient was an 83-year-old Turkish female who presented to the emergency department with an acute onset of abdominal pain, high-grade fever, nausea, and vomiting. A physical examination revealed signs of an acute abdomen, characterized by diffuse abdominal tenderness, guarding, and rebound tenderness. Preliminary investigative procedures in the laboratory revealed significantly elevated levels of leukocytes and C-reactive protein (CRP), consistent with a substantial inflammatory response. An intravenous contrast-enhanced CT scan revealed a left diaphragmatic hernia containing the stomach, spleen, transverse colon, and left colon, all of which appeared to be trapped within the hernial sac. The intravenous contrast-enhanced abdominal CT revealed the presence of free fluid in the right paracolic area and pelvis. However, no clear finding favoring the presence of free air in the herniated sac that had herniated into the thorax was identified. In view of the patient's acute abdominal findings, elevated acute-phase reactants in laboratory parameters, and data obtained from CT, this guided the emergency surgical decision. Of note, the decision to perform a splenectomy was made intraoperatively, and the preoperative imaging did not reveal any masses in the spleen.

During the operative intervention for this acute surgical emergency, both a splenic lymphangioma and an AMN were incidentally discovered, unrelated to her acute symptoms.

Surgical procedures

Upon laparotomy, a diaphragmatic defect measuring approximately 10 cm in diameter was identified in the left hemidiaphragm. The herniated abdominal viscera were meticulously reduced and examined. Multiple perforations were identified in the transverse colon, accompanied by widespread ischemia involving the terminal ileum, cecum, and right colon. Furthermore, the necessity for a splenectomy was determined due to severe ischemia and irreparable structural damage observed within the compromised hernia sac. The spleen was found to have impaired circulation and was trapped in the hernia sac. Some crucial separations were observed in the capsule of the spleen, and bleeding was evident in these areas. The decision to perform a splenectomy was made to eliminate the risk of life-threatening bleeding in the patient. The surgical procedure involved the resection of the terminal ileum, the right colon, and the transverse colon, and the splenectomy. The diaphragmatic defect was closed using continuous 0-gauge Vicryl sutures. The specimens, comprising a portion of the colon resection, splenectomy, and hernia sac, from an 83-year-old female patient who underwent operative intervention for an acute abdomen secondary to herniation, were dispatched to our pathology laboratory.

Macroscopic and microscopic histopathologic examination

**Figure 1:**
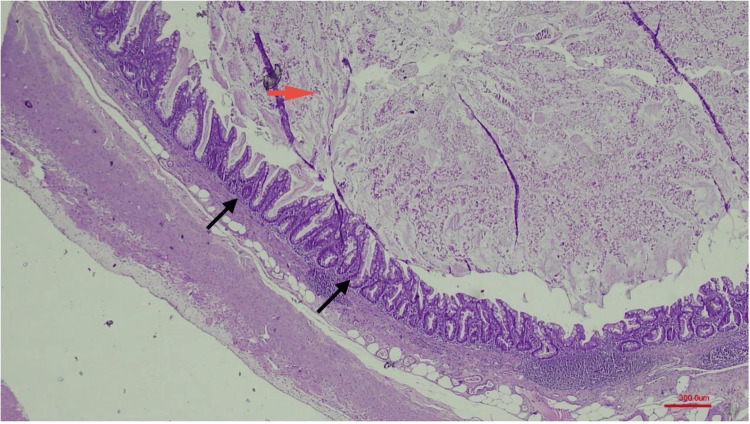
Histopathological examination of the appendix shows mucinous epithelium proliferating in a pushing pattern toward the muscular layer without intervening lamina propria, villous epithelium with low-grade cytologic atypia (black arrows), and mucin within the lumen (red arrow).

**Figure 2:**
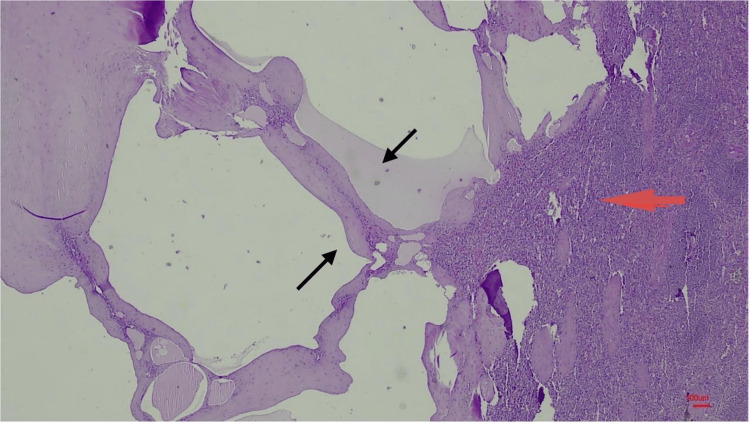
Splenic lymphangioma characterized by dilated lymphatic vessels (black arrows) within the splenic parenchyma (red arrow) (hematoxylin-eosin, 4 × 100).

**Figure 3:**
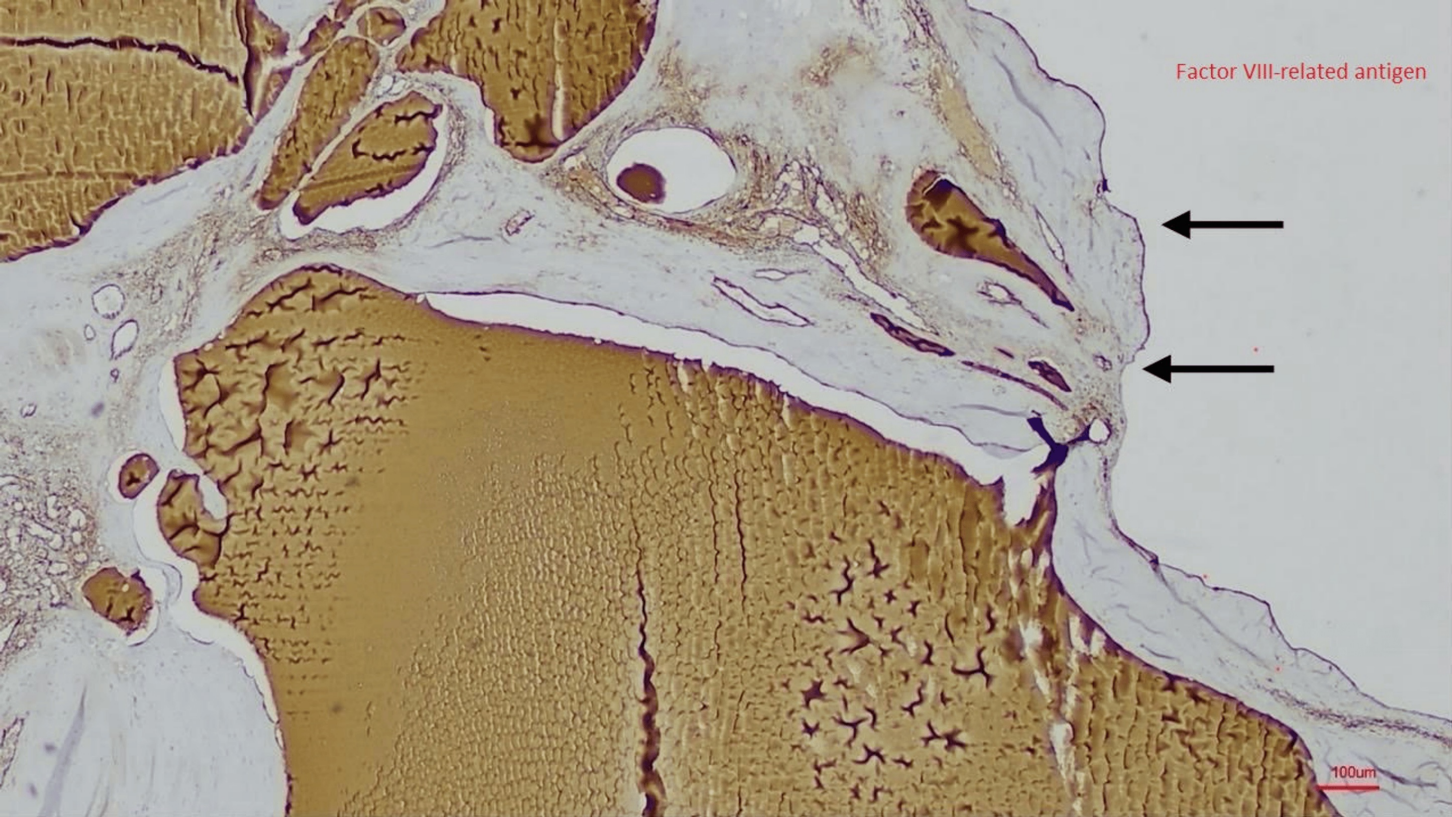
Lymphatic vessel endothelium stained with factor VIII-related antigen (factor VIII-R antigen), confirming the diagnosis of lymphangioma. The endothelial lining is visible as a linear pattern on the surface (black arrows) (factor VIII-R antigen, 4 × 100).

Upon macroscopic inspection, the colon resection material, measuring approximately 67 cm in length and including an appendix 3 cm long, revealed a multitude of diverticula. A section of the appendix displayed mucinous material, the entirety of which was submitted for examination. Furthermore, the splenectomy specimen, measuring 9.5 × 5 × 3 cm, exhibited a lesion 2.5 cm in diameter, containing cystic and calcified areas. Subsequent histopathological examination disclosed mixed-type inflammatory cell infiltration within the serosa of the bowel and the hernia sac. Low-grade mucinous neoplasm was confirmed in the appendiceal samples (Figure 1). Within the splenic lesion, cystic spaces lined by endothelial cells and filled with proteinaceous fluid were observed (Figure 2). Immunohistochemical analysis confirmed factor VIII-related antigen (factor VIII-R antigen) positivity, thereby verifying the diagnosis of splenic lymphangioma (Figure 3).

Postoperative period

In the postoperative period, the patient was admitted to the intensive care unit on a ventilator following surgery. Despite the implementation of supportive measures and aggressive medical management, progressive multi-organ failure ensued. On the fifth postoperative day, there was a further deterioration in the patient's condition, resulting in demise.

## Discussion

Lymphangiomas are infrequent congenital malformations of the lymphatic system. They are often diagnosed in childhood, while in adults, their detection is commonly incidental. Histologically, three subtypes have been delineated: capillary, cavernous, and cystic, the cystic type being the most prevalent [1,2]. Splenic lymphangiomas are rare entities, constituting less than 0.007% of all tumors, and are usually discovered by chance in adult patients. In exceptional instances, symptoms such as pain in the left upper quadrant, nausea, or anorexia may manifest, or signs of compression may be evinced [1]. In the instant case, the splenic lymphangioma was entirely an incidental discovery made during acute abdominal surgery. While imaging modalities lend assistance to diagnosis, histopathological examination remains requisite for a conclusive determination. The differential diagnosis for splenic cystic lesions encompasses hydatid cyst, hemangioma, lymphoma, metastasis, and pseudocyst.

The biological comportment and clinical course of AMNs are heterogeneous. There exists a significant number of patients presenting with manifestations of acute appendicitis, alongside those cases that remain clinically silent [6]. AMN, though rare, do represent a heterogeneous group of tumors concerning their clinical management. According to the World Health Organization (WHO) classifications, these lesions are stratified as low-grade appendiceal mucinous neoplasm (LAMN), high-grade appendiceal mucinous neoplasm (HAMN), and mucinous adenocarcinoma. In cases of LAMN, a localized disease course is typically observed, whereas the risk of developing PMP is augmented in the presence of perforation. For high-grade tumors, the risk of PMP and distant metastasis is markedly elevated. The meticulous evaluation of mucinous lesions identified during surgical procedures, along with the establishment of appropriate pathological classifications and long-term follow-up protocols, is of paramount importance for the prognosis of patients [7]. To date, no documented instance has been reported in the medical literature of splenic lymphangioma and AMN being simultaneously identified as primary lesions within the same patient. This rare coexistence may represent an incidental occurrence, or it may suggest potential shared genetic or environmental factors.

This present case report contributes to the existing literature by documenting the coeval identification of two rare primary pathologies. This extraordinary concomitance underscores the necessity for surgeons to remain vigilant for unexpected pathologies, even in emergent circumstances [8,9] such as an acute abdomen.

## Conclusions

This case report documents the first known instance of the rare co-occurrence of splenic lymphangioma and AMN, incidentally detected during emergent acute abdominal surgery. Both pathologies typically follow an asymptomatic course and are usually diagnosed incidentally, with definitive diagnosis established through histopathological examination. This unique case highlights the critical importance of thoroughly examining all abdominal organs during surgical procedures, and histopathologic examination of all organs after resection underscores the necessity of a multidisciplinary approach for evaluating rare incidental findings to ensure appropriate pathological classification and long-term follow-up protocols.
